# Genome-Wide Analysis and Functional Correlation of Tomato *JAZ* Genes Under *Tuta absoluta* Infestation and Nanoparticle-Induced Defense

**DOI:** 10.3390/insects16101046

**Published:** 2025-10-13

**Authors:** Inzamam Ul Haq, Abdul Basit, Moazam Hyder, Mirza Naveed Shahzad, Asim Abbasi, Yasir Sharif, Muhammad Adeel Ghafar, Xiangyun Cai, Nazih Y. Rebouh, Youming Hou

**Affiliations:** 1 State Key Laboratory of Agricultural and Forestry Biosecurity, Key Laboratory of Biopesticides and Chemical Biology, MOE, College of Plant Protection, Fujian Agriculture and Forestry University, Fuzhou 350002, China; 2 Department of Statistics, University of Gujrat, Gujrat 50700, Pakistan; 3 Department of Entomology, University of Agriculture, Faisalabad 38040, Pakistan; asimuaf95@gmail.com; 4 College of Agriculture, Fujian Agriculture and Forestry University, Fuzhou 350002, China; 5 Department of Environmental Management, Institute of Environmental Engineering, RUDN University, 6 Miklukho-Maklaya St., Moscow 117198, Russia

**Keywords:** jasmonate signaling, *SlJAZ* gene family, mesoporous silica nanoparticles, *Tuta absoluta*, tomato defense mechanisms

## Abstract

Tomato (*Solanum lycopersicum*) production is increasingly threatened by *Tuta absoluta*, a destructive pest that compromises yield and quality. To explore sustainable alternatives to conventional insecticides, we investigated the jasmonate-mediated defense pathway by performing a genome-wide characterization of the JAZ gene family in *S. lycopersicum*. A total of 39 *SlJAZ* genes were identified and mapped to 12 chromosomes. Detailed analysis revealed conserved motifs, diverse exon–intron structures, four major phylogenetic groups, and the presence of multiple MeJA- and stress-responsive cis-elements. Synteny analysis indicated gene duplication events and evolutionary conservation with Arabidopsis and potato. Small RNA predictions suggested that 33 *SlJAZ* genes are targeted by 69 microRNAs, implying multilayered regulation. Transcriptome analysis under four treatment conditions—mesoporous silica nanoparticles (MSNs) ± pest infestation—revealed 21 differentially expressed SlJAZ genes. *SlJAZ1*, *SlJAZ19*, *SlJAZ20*, and *SlJAZ22* were notably upregulated under the combined MSN and pest treatment, with expression patterns validated by qRT-PCR (R^2^ = 0.92). Phenotypic assessment of leaf damage index, larval survival rate, and number of leaf mines showed reduced pest activity in MSN-treated plants. Regression analysis demonstrated significant negative correlations between expression levels of *SlJAZ20*, *SlJAZ26*, and *SlJAZ29* and pest-related damage traits. These findings indicate that MSNs function as effective elicitors of JA-responsive defense in tomato and modulate the expression of specific JAZ genes linked to enhanced resistance. The study provides a valuable foundation for integrating nanotechnology with molecular defense strategies to promote sustainable pest management.

## Simple Summary

Tomato crops around the world are under serious threat from an insect pest called *Tuta absoluta*, which damages the leaves and reduces crop quality and yield. Traditional pesticides used to control this pest can harm the environment and human health. In this study, we explored a safer and more sustainable way to boost the plant’s natural defense system. We focused on a group of genes in tomato plants that help defend against insect attack. We tested the use of mesoporous silica nanoparticles, which can trigger the plant’s defense without harming the environment. By studying changes in gene activity and measuring the damage caused by insects, we found that these nanoparticles reduced pest survival and damage on the plants. Certain defense-related genes were strongly linked to better resistance. This research shows that it is possible to use eco-friendly materials to protect crops by strengthening their own defense systems. The findings could help farmers reduce pesticide use and grow healthier crops in a more sustainable way.

## 1. Introduction

Plants have developed complex defense mechanisms to protect themselves from various biotic and abiotic stresses [1]. A central component of these defense mechanisms is the jasmonic acid (JA) signaling pathway, which plays a pivotal role in regulating plant responses to herbivore attacks, pathogen infections, and environmental challenges [2]. Within this pathway, the Jasmonate ZIM-domain (JAZ) family of proteins acts as transcriptional repressors of JA-responsive genes [3]. Under non-stress conditions, JAZ proteins suppress the expression of defense genes by interacting with transcription factors such as MYC2. However, when plants encounter stress, elevated levels of JA trigger the degradation of JAZ proteins through the ubiquitin-proteasome pathway, allowing for the activation of defense-related gene expression [4,5].

The study of the JAZ gene family has garnered considerable attention due to its crucial role in orchestrating plant defense mechanisms [6,7]. *Solanum lycopersicum* (tomato), a crop of significant economic and agricultural importance, serves as an ideal model for investigating these mechanisms [8,9,10]. Tomatoes are cultivated extensively worldwide, and their susceptibility to various pests and diseases can lead to significant yield losses, directly impacting food security and economic stability in many regions [11,12]. The understanding of defense-related gene families such as the JAZ genes in tomatoes is therefore essential for developing crop varieties with enhanced resistance to stressors [13,14]. In light of growing concerns about the environmental impacts and diminishing efficacy of chemical pesticides, alternative strategies that leverage the plant’s innate defense mechanisms are increasingly needed [15,16,17,18].

Nanotechnology has emerged as a promising field in agricultural innovation, with various nanoparticles showing potential to enhance crop resilience and reduce the reliance on chemical inputs [19,20]. Mesoporous silica nanoparticles (MSNs) have attracted attention for their ability to modulate plant physiological processes and act as carriers for agrochemicals [21,22]. Their porous structure allows them to be functionalized and used as delivery systems for growth regulators, fertilizers, or pesticides [23]. In addition to enhancing plant growth, MSNs have been shown to improve tolerance to various environmental stresses by influencing gene expression and metabolic processes [24,25]. Despite the growing body of research, the specific molecular mechanisms through which MSNs affect plant defense pathways—particularly transcription factors such as the JAZ family—remain largely unexplored.

One of the most destructive pests to tomato crops is *Tuta absoluta* (Meyrick), commonly known as the tomato leaf miner [26]. Native to South America, this pest has rapidly spread across Europe, Africa, and parts of Asia, where it has caused significant damage to tomato crops in both open fields and greenhouses [27]. The feeding activity of *T. absoluta* severely reduces the photosynthetic capacity of tomato plants, leading to stunted growth, compromised fruit quality, and, in severe cases, total crop loss [28]. *T. absoluta* populations have developed resistance to several widely used insecticides, including pyrethroids and organophosphates [29,30], which has severely limited the effectiveness of conventional chemical control strategies [31]. This situation highlights the urgent need for alternative, sustainable pest control strategies that do not rely solely on chemical inputs. The use of MSNs as a tool to enhance the plant’s natural defenses against *T. absoluta* presents a promising solution. However, little is known about the combined effects of MSNs and pest infestation on gene expression, particularly in relation to defense-related genes like the JAZ gene family.

Tomato plants remain vulnerable to *T. absoluta*, and the reliance on chemical pesticides to manage this pest is becoming unsustainable due to the rapid evolution of insecticide resistance [29]. The environmental consequences of pesticide overuse further underscore the need for innovative and eco-friendly approaches to pest control [32]. Although MSNs offer a promising strategy for boosting plant defense mechanisms [33], there is limited understanding of how these nanoparticles influence the molecular responses of tomato plants, particularly under pest pressure. Specifically, the effects of MSNs on JAZ gene expression in tomatoes infested with *T. absoluta* have not been thoroughly investigated. Addressing this knowledge gap could lead to the development of new approaches that not only mitigate the damage caused by *T. absoluta* but also reduce reliance on harmful pesticides.

The primary aim of this study is to investigate the role of mesoporous silica nanoparticles in modulating the defense responses of tomato plants, with a particular focus on the JAZ gene family under infestation by *T. absoluta*. This study seeks to identify and characterize the JAZ gene family in *S. lycopersicum* through genome-wide analysis, providing a comprehensive understanding of the distribution and structure of these genes. Additionally, this research aims to evaluate the effect of MSNs on JAZ gene expression under both normal and pest-infested conditions, allowing for a comparison of molecular responses between treated and untreated plants.

## 2. Materials and Methods

### 2.1. Plant Materials and Growth Conditions

Tomato plants (*S. lycopersicum* cv. Zhefen 202), a cultivar widely grown in southeastern China, were selected for this study based on their availability in our laboratory. Seeds were germinated in sterilized soil and grown under controlled greenhouse conditions at the Plant Protection Institute. The greenhouse was maintained at 25 ± 2 °C during the day and 18 ± 2 °C at night, with a relative humidity of 60–70%. A 16 h light/8 h dark photoperiod was applied using artificial lighting to support optimal plant development. Plants were watered daily, and a standard Hoagland nutrient solution was applied weekly. At the four-leaf stage, seedlings were transplanted into 5 L pots and maintained under the same environmental conditions.

Mesoporous silica nanoparticles (MSNs), characterized by uniform pore sizes (2–50 nm), high surface area, and excellent physicochemical stability, are widely used as carriers in agricultural and biomedical applications. In this study, we utilized unloaded MSNs synthesized via the sol–gel method (see Supplementary Figure S1) to assess their direct impact on plant gene expression, eliminating the confounding effects of loaded bioactive compounds.

### 2.2. Experimental Design

For the experimental setup, tomato plants were assigned to four treatment groups based on the application of mesoporous silica nanoparticles (MSNs) and *T. absoluta* infestation: (1) WN/WI—without MSNs and without *T. absoluta* (control), (2) WN/I—without MSNs but with *T. absoluta* infestation, (3) N/WI—with MSNs but without *T. absoluta* infestation, and (4) N/I—with both MSNs and *T. absoluta* infestation. Each treatment group consisted of three biological replicates, with twenty plants per replicate, arranged in a completely randomized design.

MSNs were synthesized following the method of Chen et al. [34] and suspended in deionized water at a concentration of 30 mg/L. The suspension was applied to the foliage of N/WI and N/I plants using a handheld sprayer to ensure uniform coverage. Spraying was conducted once per week for a total of four consecutive weeks to the designated MSN treatment groups (N/WI and N/I). After the completion of the fourth spraying, neonate larvae of *T. absoluta* (<24 h old) were gently transferred onto the abaxial surface of tomato leaves using a fine brush. Each plant in the WN/I and N/I groups received 10 larvae to ensure a consistent and uniform infestation. Pest pressure was maintained throughout the experiment, and infestation levels were visually monitored daily across biological replicates to ensure homogeneity. Phenotypic parameters, including leaf damage index (LDI), larval survival rate, and the number of leaf mines, were assessed 5 days after larval infestation, similarly plant material for molecular analyses was collected 5 days after infestation.

### 2.3. Assessment of Pest-Induced Leaf Damage, Larval Survival, and Leaf Mining

To evaluate the defensive impact of mesoporous silica nanoparticles (MSNs) against *T. absoluta*, phenotypic parameters were measured across the four treatment groups. The scoring of leaf damage index (LDI) using a 0–5 ordinal scale was based on traditional approaches originally described by Hussey & Parr [35], and has been adopted in later studies [36,37]. Leaf mining activity (mines per leaf) was assessed by counting the visible mines under a stereomicroscope, following general quantification practices widely used in leaf-miner ecological studies [38].

Leaf Damage Index (LDI) was visually assessed at 5 days post-infestation on each treated plant. The extent of damage was scored using a modified 0–5 scale, where 0 = no visible damage, 1 = 1–10% leaf area damaged, 2 = 11–25%, 3 = 26–50%, 4 = 51–75%, and 5 ≥ 75%. The index for each treatment was calculated using the formula:


$$\mathrm{LDI}\;(\%)\;=\;(\frac{\sum (\mathrm{Scale}\;\times \;\mathrm{Number}\;\mathrm{of}\;\mathrm{Leaves}\;\mathrm{at}\;\mathrm{Scale})}{\mathrm{Total}\times \mathrm{Leaves}\;\mathrm{Observed}\;\times \;5})\;\times \;100$$

The average LDI was computed from five randomly selected plants per treatment.

Larval Survival Rate was calculated by counting the number of living larvae recovered from each plant at 5 days post-infestation. The survival rate was expressed as a percentage of the total number of larvae initially introduced (20 larvae per plant):


$$\mathrm{Larval}\;\mathrm{Survival}\;(\%)\;=\;(\frac{\mathrm{Number}\;\mathrm{of}\;\mathrm{Larvae}}{20})\;\times \;100$$

Leaf Mining Activity was quantified by counting the number of visible mines per leaf under a stereomicroscope. Five infested leaves per plant were randomly selected, and the total number of mines was averaged per leaf.

### 2.4. Genome-Wide Identification of JAZ Genes

The tomato reference genome (ITAG5.0) sequence was obtained from the Phytozome database (https://phytozome-next.jgi.doe.gov/ (accessed on 9 September 2024)). Candidate JAZ proteins were identified using a combination of Hidden Markov Model (HMM) searches and BLAST searches (https://blast.ncbi.nlm.nih.gov/Blast.cgi, accessed on 9 September 2024). For the HMM search, profile HMMs corresponding to the TIFY domain (Pfam ID: PF06200) and the Jas motif (Pfam ID: PF09425) were retrieved from the Pfam database (https://www.ebi.ac.uk/interpro/set/pfam/, accessed on 9 September 2024) and queried against the tomato proteome using TBtools (version 2.122) [39] with an e-value threshold of 1 × 10^−5^. *Arabidopsis* JZA proteins were downloaded from the TAIR (https://www.arabidopsis.org/index.jsp (accessed on 10 September 2024)) database. *Arabidopsis* proteins were used to BLAST against tomato protein sequences. All candidate sequences identified by HMM or BLAST were verified using Pfam, SMART, and NCBI-CDD. Genes containing both the TIFY and Jas domains were classified as canonical JAZ proteins. In line with previous reports in tomato and Arabidopsis, we also retained a subset carrying only one domain but clustering within the JAZ clade; these were designated as non-canonical JAZ-like members. All candidates were cross-checked using PlantTFDB (http://planttfdb.gao-lab.org/ (accessed on 15 September 2024)) to confirm annotation. Genes lacking both domains or those representing incomplete gene models were excluded from further analysis.

### 2.5. Determination of Physicochemical Properties

The theoretical molecular weight (MW) and isoelectric points (pI) of the proteins were analyzed using the ProtParam tool (https://web.expasy.org/protparam/; accessed on 7 October 2024) [40]. Additionally, predictions for subcellular localization were made using CELLO v2.5 (http://cello.life.nctu.edu.tw/; accessed on 24 September 2024) [41].

### 2.6. Domain and Motif Analysis

To investigate the conserved domains and motifs within the JAZ protein family in *S. lycopersicum*, a comprehensive in silico analysis was performed. Full-length amino acid sequences of the identified JAZ proteins were retrieved from the Phytozome database. The JAZ domain (Pfam ID: PF06200) and the TIFY domain (Pfam ID: PF06202) were confirmed through domain searches using the Pfam database (https://www.ebi.ac.uk/interpro/set/pfam/, accessed on 12 September 2024). Only sequences containing these domains were retained for further analysis to ensure functional relevance.

For motif discovery, the MEME Suite (v5.5.5) (http://meme-suite.org/ (accessed on 15 September 2024)) [42] was employed, with the maximum number of motifs set to 12, allowing for comprehensive identification of conserved patterns across the JAZ protein family. The identified motifs were analyzed for their distribution and conservation across different JAZ members, contributing to insights into structural variations and potential functional specialization within the family. Each motif was annotated and visualized using the MEME output, and sequences were compared across homologous JAZ proteins from other species to highlight conserved and unique structural features.

### 2.7. Phylogenetic and Gene Structure Analysis

To analyze the evolutionary relationships of the identified JAZ genes, a phylogenetic tree was constructed. Full-length amino acid sequences of the JAZ proteins from *S. lycopersicum* and homologous JAZ proteins from *Arabidopsis thaliana*, and *Solanum tuberosum* were aligned using ClustalW (version 2.1) (https://www.genome.jp/tools-bin/clustalw, accessed on 17 September 2024), and the Neighbor-Joining phylogenetic tree was generated using Poison model in MEGA X software (version 11.0.13) (https://www.megasoftware.net/, accessed on 18 September 2024) [43]. Bootstrap analysis with 1000 replicates was performed to assess the robustness of the phylogeny. Gene structure analysis was conducted by retrieving the exon-intron information of the JAZ genes from the Phytozome database, and the gene structures were visualized and mapped out on chromosomes using the TBtools software (version 2.122) [39].

### 2.8. Promoter Cis-Regulatory Elements Analysis

The promoter regions (2 kb upstream of the transcription start site) of each identified JAZ gene were analyzed to identify cis-regulatory elements associated with defense and stress responses. Promoter sequences were extracted from the *S. lycopersicum* genome. The PlantCARE database (http://bioinformatics.psb.ugent.be/webtools/plantcare/html/ (accessed on 22 September 2024)) was utilized for scanning these promoter sequences to detect putative cis-elements.

The analysis focused on identifying elements known to mediate responses to biotic and abiotic stresses, including the MeJA-responsive elements, abscisic acid-responsive elements, light-responsive elements, and other stress-associated motifs. These elements were annotated and mapped across the promoter regions of each JAZ gene, with special attention given to motifs related to jasmonate signaling, as they are critical in modulating plant defense mechanisms.

### 2.9. Gene Duplication and Synteny Analysis

The genome and GFF3 annotation files of *S. lycopersicum* were processed through MCScanX to identify duplicated gene pairs. Synonymous (Ks) and non-synonymous (Ka) substitution rates were calculated using a basic Ka/Ks calculator. The divergence time, in million years ago (MYA), was estimated for duplicated gene pairs using the formula ‘t = Ks/2r’ with a neutral substitution rate of r = 6.56 × 10^−9^ for tomato [44].

Comparative synteny analysis was conducted to investigate genome conservation between *S. lycopersicum*, *S. tuberosum*, and *Arabidopsis*, highlighting evolutionary relationships. The genome and GFF3 files were subjected to McScanX in TBtools software, and the resulting files were used for multiple synteny analysis. Homologous gene pairs were identified using MCScanX software (version 2.0), and collinear blocks were visualized to assess syntenic regions across species. Gene pairs involved in segmental or tandem duplications were highlighted to facilitate identification of duplication patterns.

### 2.10. Protein–Protein Interaction Analysis

The potential interactions of SlJAZ proteins with other proteins were analyzed by constructing a protein–protein interaction network based on their homologs in Arabidopsis, utilizing the STRING 11.5 tool (https://www.string-db.org/cgi/; accessed on 9 October 2024). Interacting proteins with maximum similarity and an E-value below 10^−5^ were selected. The top 10 interactions, filtered at a high confidence threshold of 0.7, were included in the analysis. MCL clustering was applied with an inflation parameter of 3, and dotted lines were used to represent connections between clusters.

### 2.11. Prediction of microRNAs

MicroRNA (miRNA) target prediction for JAZ genes in *S. lycopersicum* was conducted to identify potential post-transcriptional regulatory elements. The CDSs of JAZ genes were used as input in psRNATarget (https://www.zhaolab.org/psRNATarget/ (accessed on 29 September 2024)) [45] to predict miRNA binding sites. Default parameters were applied for target accessibility, with the expectation of pairing complementarity, and target sites were screened for high complementarity scores. Identified miRNA binding sites were mapped to both coding sequences (CDS) and untranslated regions (UTRs) of the JAZ genes. Only miRNAs with high predicted binding scores were retained for further analysis.

### 2.12. Gene Expression Analysis

Total RNA was extracted from tomato leaves of all four treatment groups (WN/WI, WN/I, N/WI, and N/I) using the TRIzol reagent (Invitrogen, Carlsbad, CA, USA) following the manufacturer’s protocol. RNA integrity was assessed using the Agilent 2100 Bioanalyzer (Agilent Technologies, Santa Clara, CA, USA), and samples with an RNA integrity number (RIN) ≥ 7.0 were used for library construction. RNA-seq libraries were prepared using the Illumina TruSeq RNA Sample Preparation Kit (Illumina, San Diego, CA, USA), according to the manufacturer’s instructions. mRNA was enriched using oligo(dT) magnetic beads, fragmented into ~200 bp fragments, and reverse transcribed into cDNA. After end repair, adaptor ligation, and PCR amplification, libraries were quality checked on the Agilent Bioanalyzer. Sequencing was performed on the Illumina NovaSeq 6000 platform (Illumina, USA), generating 150 bp paired-end reads. Raw reads were quality-checked using FastQC (v0.11.9) and filtered with Trimmomatic (v0.39) to remove adapters and low-quality bases. Clean reads were mapped to the tomato reference genome (ITAG5.0) using HISAT2 (v2.2.1) with default parameters. Transcript abundance was estimated as FPKM (Fragments Per Kilobase of transcript per Million mapped reads) using StringTie (v2.1.5). Differential expression analysis was conducted with DESeq2 (v1.34.0), applying a Benjamini–Hochberg adjusted *p*-value < 0.05 and |log_2_ fold change| ≥ 1 as the significance threshold. Genes meeting these criteria were considered differentially expressed. Heatmaps of expression patterns were generated using the pheatmap package in R (v4.1.0). Based on the heatmap, we selected the candidate genes to further study their expression by qPCR analysis.

### 2.13. qPCR-Based Expression Analysis

Total RNA was extracted from leaf samples of the tomato plants using the TRIzol reagent (Invitrogen, USA) according to the manufacturer’s instructions. The quality and quantity of RNA were determined using a NanoDrop spectrophotometer (Thermo Fisher Scientific, Waltham, MA, USA), and RNA integrity was verified by gel electrophoresis. First-strand cDNA was synthesized from 1 µg of total RNA using the Evo M-MLV RT Kit (Accurate Biotechnology, Hunan, Co., Ltd., Changsha, China) following the manufacturer’s protocol. qRT-PCR was performed with specific primers (Supplementary Table S1) for the selected JAZ genes (designed using Primer3 software), and the specificity of each primer pair was verified through melting curve analysis. qPCR reactions were conducted using the SYBR Green Master Mix (Bio-Rad, Hercules, CA, USA) on a CFX96 Touch Real-Time PCR Detection System (Bio-Rad, USA). The thermal cycling conditions were as follows: initial denaturation at 95 °C for 3 min, followed by 40 cycles of 95 °C for 15 s and 60 °C for 30 s. The expression levels were normalized to the tomato actin gene as an internal control. Relative expression levels were calculated using the 2^−ΔΔCt^ method, and each reaction was performed in triplicate.

### 2.14. Statistical Analysis

All experimental data were subjected to statistical analysis using R software (version 4.1.0). Data were analyzed using one-way analysis of variance (ANOVA), followed by Tukey’s post hoc test to compare differences between treatment groups. For gene expression analysis, DESeq2 was used to identify differentially expressed genes, with significance determined by an adjusted *p*-value < 0.05. qPCR data were analyzed using the same statistical methods. Regression analysis was conducted to assess the relationship between *SlJAZ* gene expression and pest-related traits (leaf damage index, larval survival rate, and number of leaf mines), with R^2^, slope, and *p*-values calculated using linear models. Results are presented as mean ± standard error, with statistical significance set at *p* < 0.05

## 3. Results

### 3.1. Identification and Distribution of JAZ Genes

**Figure 1:**
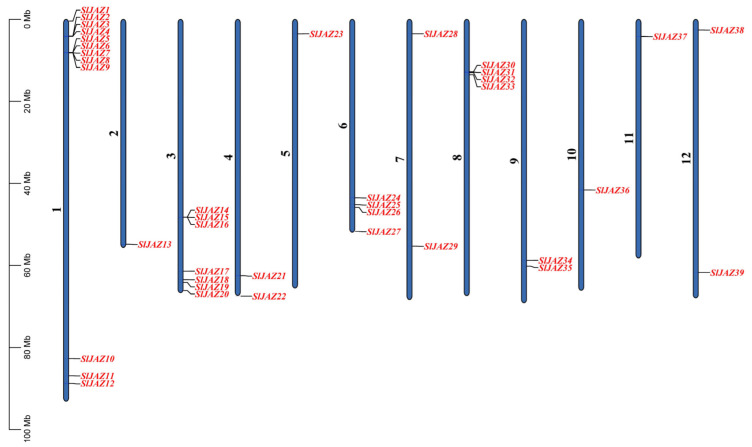
Chromosomal distribution of JAZ genes in *S. lycopersicum*. This figure shows the mapping of 39 identified JAZ genes across the 12 chromosomes of the tomato genome.

A total of 39 JAZ genes were identified in the *S. lycopersicum* genome and were mapped onto all 12 chromosomes. The chromosomal distribution of these genes is shown in Figure 1. Chromosome 1 contains the highest number of JAZ genes, with 12 genes (*SlJAZ1*, *SlJAZ2*, *SlJAZ3*, *SlJAZ4*, *SlJAZ5*, *SlJAZ6*, *SlJAZ7*, *SlJAZ8*, *SlJAZ9*, *SlJAZ10*, *SlJAZ11*, *SlJAZ12*) located between 0 Mb and 80 Mb. Chromosome 3 houses 7 genes (*SlJAZ14*, *SlJAZ15*, *SlJAZ16*, *SlJAZ17*, *SlJAZ18*, *SlJAZ19*, *SlJAZ20*), distributed along the chromosome from 10 Mb to 60 Mb.

Chromosome 4 contains 2 JAZ genes (*SlJAZ21*, *SlJAZ22*), while chromosome 2 (*SlJAZ13*), Chromosomes 5 (*SlJAZ23*), Chromosomes 10 (*SlJAZ36*) and Chromosomes 11 (*SlJAZ37*) harbors only 1 gene each. Chromosome 6 holds 4 JAZ genes (*SlJAZ24*, *SlJAZ25*, *SlJAZ26*, *SlJAZ27*), while chromosome 7 includes 2 JAZ genes (*SlJAZ28*, *SlJAZ29*). Chromosome 8 houses 4 genes (*SlJAZ30*, *SlJAZ31*, *SlJAZ32*, *and SlJAZ33*), spread between 20 Mb and 60 Mb. Finally, chromosomes 12 contains 2 JAZ genes (*SlJAZ38* and *SlJAZ39*). This distribution highlights that while some chromosomes have a higher density of JAZ genes, others have only one or two. Figure 1 and Supplementary Table S2 provides the detailed chromosomal locations of all identified genes.

### 3.2. Physicochemical Properties of SlJAZ Genes

The *SlJAZ* genes showed diverse physicochemical properties. *SlJAZ5* was the smallest gene comprising 183 bps, while *SlJAZ27* was the most extended gene with 2106 bps. Same genes showed smallest and larges proteins 61 and 702 amino acid residues. Number of exons varied from 24 in SlJAZ4 to 1 in SlJAZ34. Theoretical isoelectric points varied from 5.17 for SlJAZ19 to 10.34 for SlJAZ10. The expected molecular weight ranged between 6989.3 for SlJAZ5 to 78205.24 dalton (Da) for SlJAZ27. Grand Average of Hydropathy (GRAVY) value for protein sequences ranged from −0.954 for SlJAZ13 to 0.514 for SlJAZ22. The subcellular localization prediction showed that SlJAZ proteins are located in nucleus, mitochondria, plasma membrane, and chloroplast. Majority of the proteins were localized in nucleus, while some are located in more than one cell compartments (Supplementary Table S2).

### 3.3. Phylogenetic Analysis

**Figure 2:**
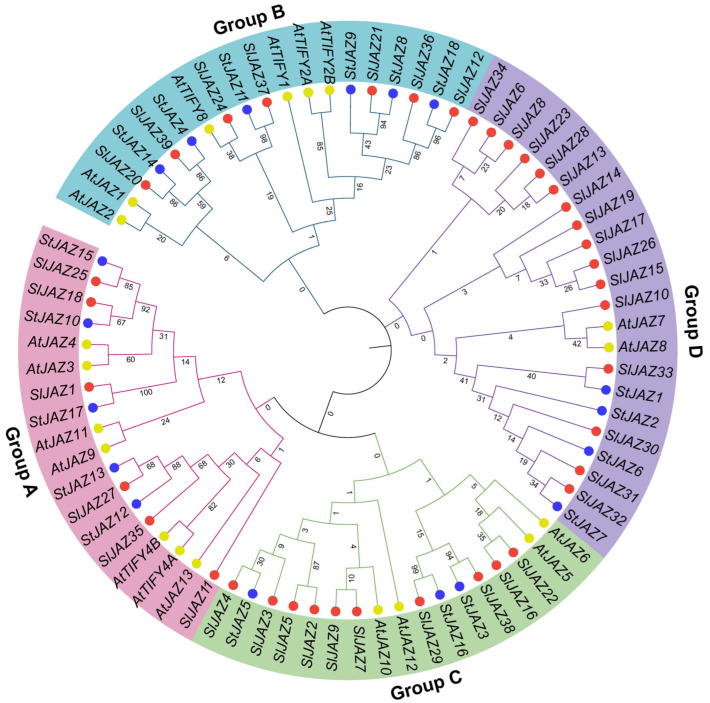
Phylogenetic relationships of JAZ proteins from *Solanum lycopersicum* (SlJAZ), *Arabidopsis thaliana* (AtJAZ), and *Solanum tuberosum* (StJAZ). The tree was constructed using the maximum likelihood method with 1000 bootstrap replicates and classified the 39 *SlJAZ* proteins into four major groups (A–D). Homologous proteins from *A. thaliana* and *S. tuberosum* were included for comparative analysis. Distinct background shading indicates the four groups, while node colors represent species origin: red = *S. lycopersicum*, blue = *S. tuberosum*, and yellow-green = *A. thaliana*. Grouping patterns supported by high bootstrap values highlight conserved evolutionary relationships, species-specific expansions, and functional conservation within the JAZ family.

Figure 2 illustrates the phylogenetic relationships among JAZ proteins from *Arabidopsis thaliana* (AtJAZ), *Solanum lycopersicum* (SlJAZ), and *Solanum tuberosum* (StJAZ), which were grouped into four distinct clades (A–D). Genes are color-coded by species, and background shading highlights the four groups. Group A consisted of 18 members, including *StJAZ15*, *SlJAZ25*, *SlJAZ18*, and *AtJAZ3*, with several clusters supported by high bootstrap values (>80). Members of this clade retained the canonical ZIM and Jas domains, suggesting strong structural conservation. Group B comprised 19 genes, such as *AtTIFY2B*, *SlJAZ20*, and *SlJAZ37*. Compared with Groups A and C, Group B showed longer branch lengths, indicating an earlier divergence. Several members exhibited motif variations, particularly in the Jas domain, and were uniquely predicted to contain miRNA binding sites such as miR164 and miR482.

Group C, containing 17 genes including *SlJAZ4*, *AtJAZ10*, and *StJAZ16*, displayed shorter branch lengths and tight clustering, consistent with a more recent divergence. Members of this clade carried highly conserved ZIM and Jas domains and were enriched in stress-responsive cis-elements in their promoter regions. Group D was the largest clade, encompassing 22 members, predominantly from tomato. This expansion suggests species-specific duplication within *S. lycopersicum*. While Group D proteins retained both conserved domains, variation in promoter cis-elements and the absence of predicted miR319 target sites in some members indicated potential diversification in regulatory functions.

### 3.4. Motif, Gene Structure, and Domain Analysis

**Figure 3:**
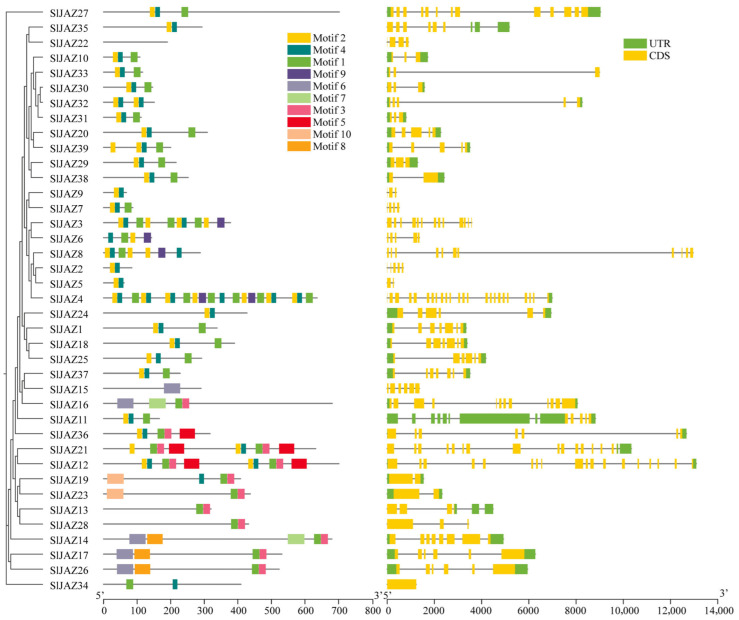
Conserved motifs in JAZ proteins of *S. lycopersicum***.** This figure illustrates the distribution of 10 conserved motifs identified across the 39 JAZ proteins in tomato. Motifs 1 and 2, present in nearly all JAZ proteins, highlight their critical roles in maintaining core functions within the jasmonate signaling pathway. Variations in motif composition among JAZ family members suggest functional diversification, contributing to specialized roles within the plant’s defense responses.

The motif and gene structure analysis of the 39 identified JAZ genes in *S. lycopersicum* revealed a diverse array of conserved motifs and gene structures associated with their roles in jasmonate signaling (Figure 3, Supplementary Table S3). In total, 12 distinct motifs were detected, with motif 7 being the longest (50 amino acids) and motifs 2 and 4 the shortest (15 amino acids each). Among them, motifs 1 and 2 were the most conserved and were present in the majority of JAZ proteins, underscoring their essential role in maintaining the core structure and function of these proteins. However, a few exceptions were observed: motif 1 was absent in *SlJAZ21*, *SlJAZ19*, and *SlJAZ12*, while motif 2 was missing in *SlJAZ21*, *SlJAZ19*, *SlJAZ12*, and *SlJAZ36*. Motif 1 corresponds to the conserved TIFY/ZIM domain, which is critical for protein–protein interactions and recruitment of the NINJA–TPL complex, whereas motif 2 represents the Jas domain, which mediates binding to the SCF^COI1 complex for jasmonate-dependent degradation of JAZ repressors. The absence of these motifs in specific members may reflect functional divergence within the family. Other motifs (3–10) were found in a subset of proteins and may contribute to subgroup-specific specialization.

**Figure 4:**
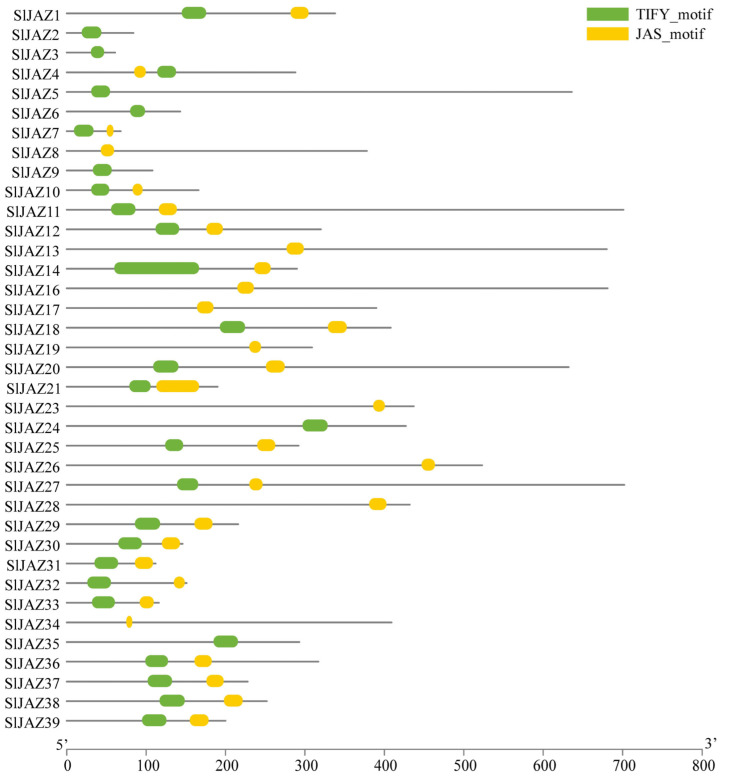
Gene structure and conserved domains of JAZ genes in *S. lycopersicum*. This figure displays the exon-intron organization and conserved domains (TIFY and JAS) within the 39 JAZ genes. Variability in gene structures, with differences in the number and length of exons and introns, suggests functional diversity among JAZ genes. The presence of the conserved TIFY domain (green) across all genes and the JAS domain (yellow) in most genes highlights their essential roles in jasmonate-mediated signaling and protein interactions.

The gene structure analysis, as shown in Figure 4, revealed considerable variation in exon-intron organization among the JAZ genes. While some genes, such as *SlJAZ6* and *SlJAZ23*, exhibited a relatively simple structure with a few exons and introns, other genes, such as *SlJAZ21* and *SlJAZ36*, displayed more complex gene structures with multiple exons and introns. Additionally, the length of untranslated regions (UTRs) varied significantly among the JAZ genes, which may play a role in the post-transcriptional regulation of these genes under different stress conditions. The domain analysis further highlighted the presence of the TIFY and JAS domains, which are essential for the functioning of JAZ proteins in jasmonate signaling. Figure 4 shows that all 39 JAZ proteins contain the conserved TIFY domain (green) necessary for protein–protein interactions, and most of the JAZ proteins also possess the JAS domain (yellow), which is required for interaction with the COI1 receptor. These domains are highly conserved across the JAZ proteins, reflecting their fundamental role in jasmonate-mediated defense responses. The comparison of gene structures and motifs across the JAZ gene family suggests both conserved functions and potential specialization among the different members.

### 3.5. Cis-Regulatory Elements

**Figure 5:**
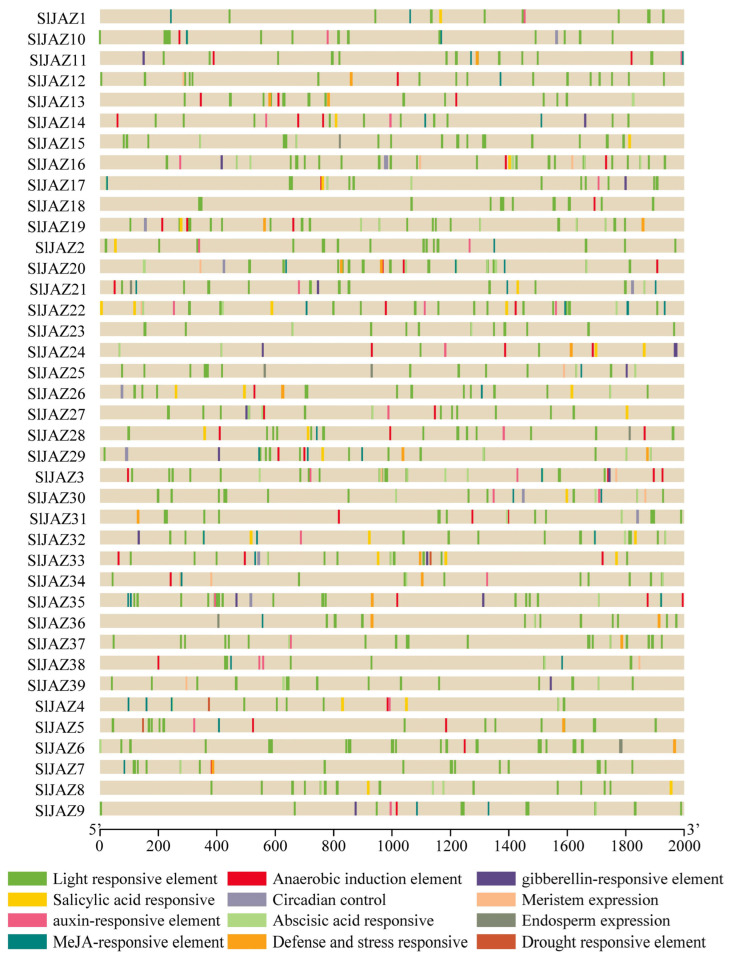
Cis-regulatory elements in promoter regions of JAZ genes in *S. lycopersicum*.

The analysis of the cis-regulatory elements in the promoter regions of the 39 JAZ genes revealed a wide range of elements associated with various stress responses, hormonal regulation, and development. As shown in Figure 5, the promoter regions (2 kb upstream of the transcription start site) contained numerous light-responsive elements, which were the most abundant across all JAZ genes, suggesting that light regulation may play a significant role in the expression of these genes. Additionally, several stress-responsive elements, such as the MeJA-responsive elements, abscisic acid-responsive elements, and defense and stress-responsive elements, were identified in the majority of the JAZ genes.

Salicylic acid-responsive elements, anaerobic induction elements, and auxin-responsive elements were also prevalent, indicating a potential role for these genes in responding to biotic and abiotic stresses through hormonal signaling pathways. Gibberellin-responsive elements, drought-responsive elements, and circadian control elements were present in several JAZ genes, suggesting that these genes might be involved in developmental processes and environmental stress responses. Furthermore, cis-elements related to meristem expression, endosperm expression, and anaerobic induction were less frequent but still observed in a subset of the JAZ genes, indicating specialized regulatory roles in plant growth and development. Overall, the presence of a diverse array of cis-regulatory elements in the promoter regions of the JAZ genes highlights their potential involvement in a wide range of regulatory processes, particularly in response to environmental stimuli and hormonal signals.

### 3.6. Duplication and Synteny Analysis

**Figure 6:**
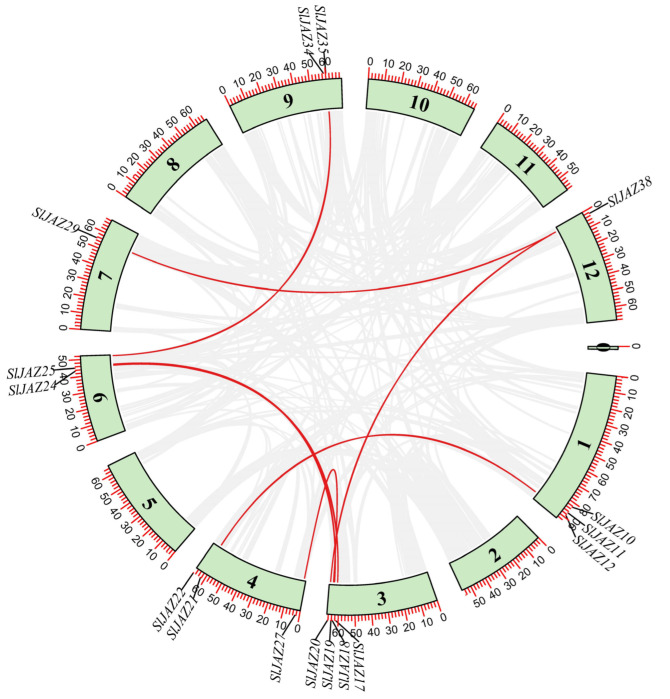
Chromosomal location and duplication events of JAZ genes in *S. lycopersicum*. *SlJAZ* genes predominantly shows segmental duplication.

The duplication analysis revealed seven duplicated gene pairs (Table 1, Figure 6) out of 39 *SlJAZ* genes. One gene pair (*SlJAZ19-SlJAZ20*) was tandemly duplicated, while all others showed segmental duplication. These segmental duplications suggest that gene duplication has played a critical role in the expansion of the JAZ gene family in tomato. The Ka/Ks ratios of duplicated *SlJAZ* gene pairs were all less than one, indicating that although duplication events are stochastic in origin, purifying selection has subsequently acted on these gene pairs to preserve their functions and maintain evolutionary stability. The estimated duplication times ranged from 43.63 million years ago (MYA) for *SlJAZ17-SlJAZ26* to 144.04 MYA for *SlJAZ12-SlJAZ21* (Table 1). Figure 6 shows the duplicated gene pairs on different chromosomes of *S. lycopersicum*; the red lines shows the duplicated genes, while gray lines in the background shows the duplication blocks of whole genome.

**Figure 7:**
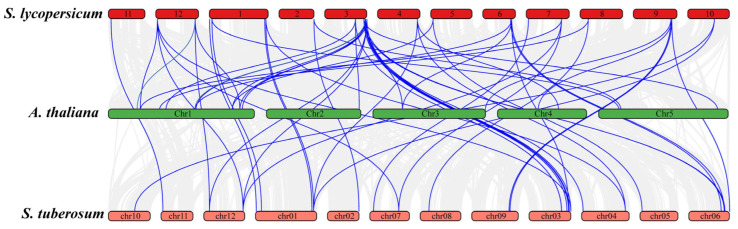
Synteny analysis of JAZ genes between *S. lycopersicum*, *S. tuberosum*, and *A. thaliana*. *S. lycopersicum* and *S. tuberosum* shred more syntenic genes as compared to *S. lycopersicum* and *Arabidopsis*.

In addition to the duplication events within *S. lycopersicum*, Figure 7 highlights the synteny relationships of JAZ genes between *S. lycopersicum*, *A. thaliana*, and *S. tuberosum*. Numerous syntenic blocks were observed between *S. lycopersicum* and *S. tuberosum*, and *A. thaliana*, which is expected due to their shared evolutionary history. *S. lycopersicum* shared 15 syntenic JAZ genes with *Arabidopsis*, while the syntenic genes among *S. lycopersicum* and *S. tuberosum* were 20 (Figure 7). Higher number of syntenic genes among *S. lycopersicum* and *S. tuberosum* showed there more closed evolutionary relationships compared to *Arabidopsis*, which reflects more distant evolutionary relationship between these species. The identification of these syntenic relationships and duplication events is significant in understanding the evolutionary history of the JAZ gene family.

### 3.7. miRNA Prediction

**Figure 8:**
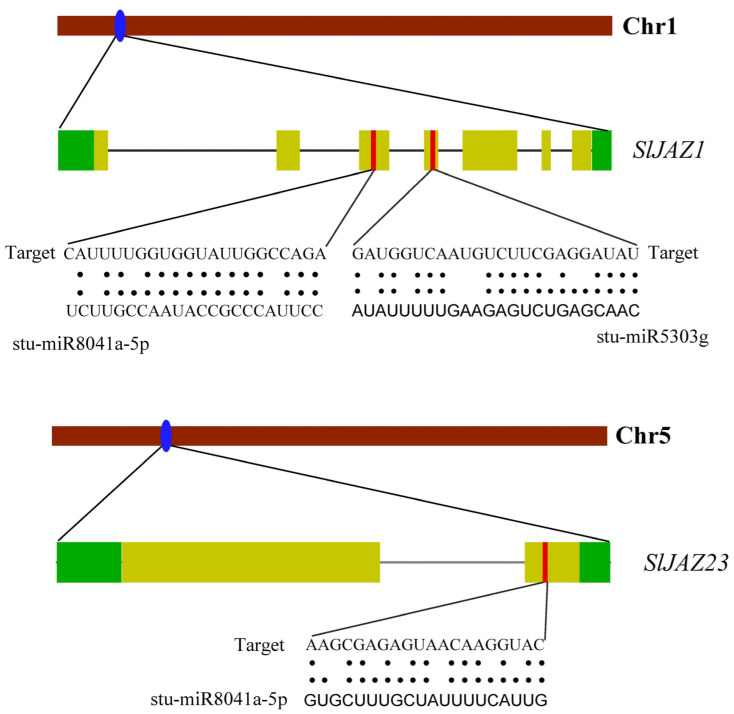
Predicted miRNA target sites in JAZ genes of *S. lycopersicum*. This figure highlights the miRNA binding sites predicted for JAZ genes, focusing on *SlJAZ1* and *SlJAZ23* as potential targets.

The miRNA prediction analysis identified several miRNAs that may regulate the expression of JAZ genes in *S. lycopersicum*. Overall, 69 miRNAs from 39 families targeted 33 genes (Supplementary Table S4). *SlJAZ25* was targeted by most of the miRNAs, followed by *SlJAZ18*, *SlJAZ12*, and *SlJAZ4*, and so on. Similarly, stu-miR8029, stu-miR8037, stu-miR169f-5p, stu-miR169g, targeted the greatest number of genes (Supplementary Table S4). Schematic representation of miRNAs, target genes, and target sites is given in Figure 8. Specifically, *SlJAZ1*, located on chromosome 1, was predicted to be targeted by two miRNAs: stu-miR8041a-5p and stu-miR5303g. These miRNAs target regions in the coding sequence of *SlJAZ1*, suggesting that they may play a role in post-transcriptional regulation by reducing the stability or translational efficiency of the JAZ transcripts. Similarly, *SlJAZ23*, located on chromosome 5, was also predicted to be targeted by stu-miR8041a-5p, indicating that this miRNA may have multiple regulatory targets within the JAZ gene family. The predicted miRNA binding sites were found in both the 3′ untranslated regions (UTRs) and the coding regions of the JAZ genes, suggesting diverse mechanisms of miRNA-mediated regulation.

### 3.8. Expression Profiling of SlJAZ Genes Under Different Treatments

**Figure 9:**
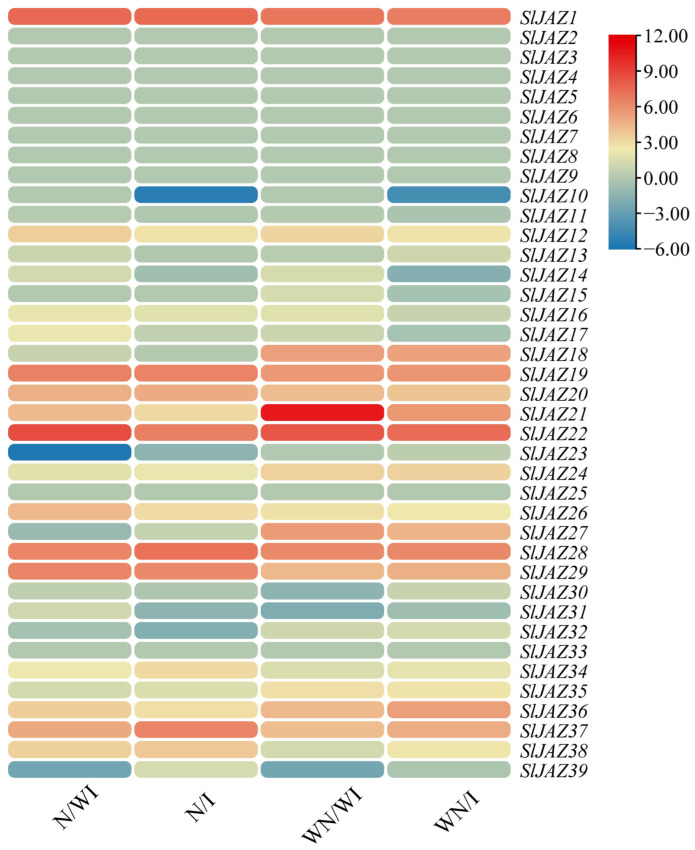
Expression profiling of selected *SlJAZ* genes under different treatments. Heatmaps represent relative expression patterns across the four treatment groups: WN/WI (without MSNs, without infestation), WN/I (without MSNs, with infestation), N/WI (with MSNs, without infestation), and N/I (with MSNs, with infestation). Expression values were log_2_-transformed and normalized, with red indicating upregulation and blue indicating downregulation.

The global expression profiles of the 39 *S. lycopersicum* JAZ genes were analyzed across four treatment groups: N/WI (with nanoparticles, without infestation), N/I (with nanoparticles, with infestation), WN/WI (without nanoparticles, without infestation), and WN/I (without nanoparticles, with infestation). The results, visualized in Figure 9 using a heatmap, reveal distinct expression patterns of the JAZ genes in response to the different treatments. Several JAZ genes showed significant upregulation or downregulation under specific treatments. For instance, *SlJAZ9* and *SlJAZ21* exhibited notable downregulation in the N/WI treatment, while *SlJAZ20* and *SlJAZ22* were highly upregulated in the N/I treatment. In the WN/I group, *SlJAZ9* was downregulated, indicating a possible role in pest stress responses without the influence of nanoparticles. Additionally, *SlJAZ1*, *SlJAZ2*, *SlJAZ3*, *SlJAZ19*, and *SlJAZ22* showed consistent upregulation in response to *T. absoluta* infestation, particularly in the presence of nanoparticles (N/I).

*SlJAZ1*, *SlJAZ19*, *SlJAZ20*, and *SlJAZ22* showed a distinct expression pattern, being significantly upregulated only under the combined treatment of MSNs and *T. absoluta*. This suggests that MSNs may function as priming agents, enhancing the jasmonate pathway to enable a faster or stronger response upon pest attack. The lack of similar upregulation in the MSNs-only or pest-only treatments further indicates that these genes may respond specifically to stress integration rather than individual stimuli. Additionally, the downregulation of *SlJAZ11* and *SlJAZ30* in pest-infested plants without MSNs but not in those pretreated with MSNs implies that nanoparticle exposure may help maintain regulatory homeostasis in jasmonate signalling under biotic stress; however, further studies are warranted to substantiate this role of MSNs and to clarify the underlying regulatory mechanisms.

### 3.9. Phenotypic Effects of Tuta absoluta and MSNs on Tomato Plants

To evaluate the role of mesoporous silica nanoparticles (MSNs) in modulating tomato plant resistance against *Tuta absoluta*, we assessed three key phenotypic traits: leaf damage index, larval survival rate, and the number of leaf mines (Table 2). In *T. absoluta*–infested plants without MSN treatment (WN/I), the leaf damage index reached 45.2 ± 4.7%, which was significantly higher than the 20.6 ± 3.8% observed in the MSN-treated infested group (N/I) (F(3,16) = 92.41, *p* < 0.01). No visible damage was recorded in the control (WN/WI) or MSN-only (N/WI) groups. Larval survival was also significantly reduced by MSN application. The WN/I group exhibited 84.6 ± 2.9% larval survival, while the N/I treatment led to a marked decrease to 52.3 ± 4.4% (F(3,16) = 77.86, *p* < 0.01). The number of leaf mines per leaf declined from 12.4 ± 1.8 in WN/I to 5.1 ± 1.2 in N/I (F(3,16) = 68.13, *p* < 0.01), confirming a significant reduction in feeding activity under nanoparticle exposure. No mines were observed in WN/WI or N/WI treatments. Overall, the application of MSNs significantly suppressed pest-induced leaf damage, reduced larval viability, and decreased mining activity, indicating enhanced defense performance under pest pressure.

### 3.10. Regression Analysis of SlJAZ Gene Expression and Pest-Related Parameters

Regression analysis was conducted to evaluate the relationship between the relative expression levels of 13 *SlJAZ* genes and three pest-related phenotypic parameters: leaf damage index, larval survival rate, and number of leaf mines. The analysis revealed gene-specific variations in the strength and direction of association with each trait. For leaf damage index, *SlJAZ20* showed the highest coefficient of determination (R^2^ = 0.831, *p* = 0.090), followed by *SlJAZ29* (R^2^ = 0.579, *p* = 0.281) and *SlJAZ26* (R^2^ = 0.462, *p* = 0.365). Similarly, negative correlations were observed between *SlJAZ20*, *SlJAZ26*, and *SlJAZ29* expression and the number of leaf mines, with *SlJAZ20* again exhibiting the strongest correlation (R^2^ = 0.831). Regression coefficients for genes such as *SlJAZ31*, *SlJAZ39*, and *SlJAZ10* remained low (R^2^ < 0.2) across all traits, indicating weak associations. For larval survival rate, valid regression could only be performed for treatments with complete data. Among these, *SlJAZ20* and *SlJAZ29* yielded the highest R^2^ values (0.923 and 0.853, respectively), indicating potential associations under specific conditions (Supplementary Table S5).

### 3.11. qPCR Validation

**Figure 10:**
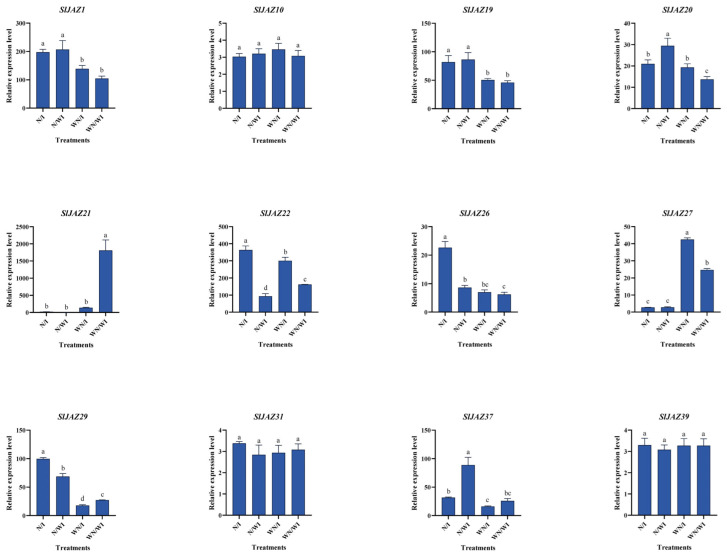
Validation of JAZ gene expression by qPCR in *S. lycopersicum* under different treatments. This figure confirms RNA-seq expression trends for selected JAZ genes across the four treatment groups: N/WI (nanoparticles without infestation), N/I (nanoparticles with infestation), WN/WI (no nanoparticles, no infestation), and WN/I (no nanoparticles, with infestation) using quantitative real-time PCR. Different lowercase letters (a–d) above the bars indicate statistically significant differences among treatments according to one-way ANOVA followed by Tukey’s HSD test (*p* < 0.05).

Quantitative real-time PCR (qPCR) was performed to validate the RNA-seq results for a subset of 12 *SlJAZ* genes that showed significant differential expression across the four treatment groups: WN/WI (without nanoparticles, without infestation), WN/I (without nanoparticles, with infestation), N/WI (with nanoparticles, without infestation), and N/I (with nanoparticles, with infestation). The relative expression levels (Figure 10) were largely consistent with RNA-seq data and highlighted three distinct trends. The comparison of WN/WI with WN/I revealed transcriptional responses to pest infestation. *SlJAZ21* was strongly downregulated under infestation, while *SlJAZ22* showed moderate induction, suggesting divergent roles in basal defense signaling. Several other genes, including *SlJAZ1*, *SlJAZ19*, and *SlJAZ20*, were also significantly induced by infestation. The comparison of WN/WI with N/WI showed that MSN treatment alone was sufficient to trigger the upregulation of multiple genes, including *SlJAZ21* and *SlJAZ22*. This suggests that nanoparticles may act as priming agents, enhancing the readiness of JA signaling components even in the absence of biotic stress. Under combined treatment (N/I), several genes displayed expression patterns that differed from those observed under either stress or nanoparticle exposure alone. Notably, the downregulation of *SlJAZ21* observed in WN/I was reversed under N/I, while *SlJAZ22* exhibited further induction. Similarly, *SlJAZ27* and *SlJAZ37* showed their highest expression in the N/I group. These results indicate that MSN pretreatment not only modifies the expression of individual JAZ genes but also influences their regulatory behavior under pest challenge.

## 4. Discussion

The JAZ gene family in *S. lycopersicum* is integral to jasmonate (JA) signaling, a pathway critical for coordinating defense responses against a wide range of biotic stresses [5,46]. JAZ proteins act as transcriptional repressors, preventing the activation of JA-responsive genes under normal conditions. When plants encounter stress, JA levels rise, triggering the ubiquitination and degradation of JAZ proteins, thereby relieving their repression on transcription factors like MYC2, which initiates the expression of defense-related genes [4,47]. Our results align with this model, demonstrating that several JAZ genes in tomato, including *SlJAZ1*, *SlJAZ9*, and *SlJAZ19*, respond to pest and nanoparticle treatments through differential expression. These findings mirror observations by Campos et al. [48], who reported that specific JAZ genes in tomato are selectively activated in response to stress, underscoring their specialized roles within the plant’s defense network. Compared with the earlier report by Sun et al. [49] which identified 26 *SlJAZ* genes and Thines et al. [50], who reported 17 genes, our analysis revealed 39 members in tomato. This expansion was made possible by the use of updated genome annotation combined with advanced domain- and profile-based searches using the Pfam database, SMART, and HMMER3.0. Integration of these tools allowed us to detect additional low-similarity sequences carrying the conserved TIFY and/or Jas domains, thereby expanding the repertoire of JAZ genes identified in tomato.

Moreover, phylogenetic and structural analyses of JAZ genes suggest that these genes have diversified to fulfill specialized functions across species [51,52]. In tomato, *SlJAZ21* and *SlJAZ22* exhibited contrasting expression responses; *SlJAZ21* was suppressed by infestation but restored under MSN treatment, whereas *SlJAZ22* was consistently induced, suggesting functional divergence between these two closely related members. Similar findings in other plant species, such as *A. thaliana* and *O. sativa*, reveal that JAZ genes have undergone adaptive evolution, equipping plants with finely tuned responses to environmental stressors [3,50]. These comparisons highlight that while the core functions of JAZ proteins in JA signaling are conserved, the gene family’s diversity allows for flexible and specific responses, a feature that could be exploited in breeding for enhanced pest resistance.

Nanotechnology has emerged as an innovative approach to enhance plant resilience, and mesoporous silica nanoparticles (MSNs) have shown potential in modulating plant physiology [25,53]. In this study, MSNs were applied to tomato plants to examine their effects on JAZ gene expression, revealing significant upregulation of genes such as *SlJAZ20* and *SlJAZ22*. These results are consistent with previous research by Torabian, Zahedi and Khoshgoftar [25], who found that nanoparticles can induce stress tolerance mechanisms by modulating gene expression, particularly under abiotic stresses such as salinity. MSNs are thought to enhance defense pathways by acting as elicitors, potentially priming the plant’s defense machinery through interaction with key regulatory pathways like JA signaling [54]. The upregulation of *SlJAZ20* and *SlJAZ22* suggests that MSNs may influence JA signaling, a mechanism previously observed in studies on nanoparticle-mediated priming of defense responses in wheat and rice [55].

While MSNs have been widely studied for their ability to deliver agrochemicals and enhance nutrient availability [56], their direct influence on transcriptional regulation in defense pathways is relatively novel. Here, MSNs may have induced a “primed” state, wherein the JA pathway is activated upon the onset of pest pressure, providing plants with a rapid and robust defense response. This mechanism aligns with studies on silicon nanoparticles in plants, which have shown that nanoparticles can activate antioxidant and other defense-related genes [57,58]. Consequently, MSNs offer a promising alternative to traditional chemical pesticides by activating innate defense pathways and enhancing resilience, reducing environmental toxicity concerns [59].

*T. absoluta*, commonly known as the tomato leaf miner, is a significant pest of tomato crops worldwide, and managing this pest has become increasingly challenging due to its resistance to chemical pesticides [27,60]. The current study shows that *T. absoluta* infestation significantly alters JAZ gene expression in tomato plants, with distinct expression patterns observed in tolerant and susceptible plants. In untreated plants under infestation (WN/I), *SlJAZ9* and *SlJAZ21* were significantly downregulated, suggesting that pest-induced stress can suppress specific components of the JA pathway. Such suppression has been associated with increased susceptibility in previous studies [47]. Conversely, genes such as *SlJAZ1* and *SlJAZ19* were upregulated, potentially reflecting an attempt by the plant to mount a defense response.

Our findings further indicate that MSNs may have a protective effect, as evidenced by higher expression levels of several JAZ genes in MSN-treated plants under infestation (N/I). This observation supports studies by Raliya, Nair, Chavalmane, Wang and Biswas [57] and Sun, Hussain, Yi, Rookes, Kong and Cahill [56], which suggest that nanoparticle application can enhance defense responses under biotic stress conditions. The expression of JAZ genes, particularly *SlJAZ1*, *SlJAZ2*, and *SlJAZ3*, in the presence of MSNs suggests that these nanoparticles might mitigate pest-induced stress, enhancing plant resilience. This response aligns with recent work showing that nanoparticle-treated plants often exhibit upregulated antioxidant and defense pathways [55,58], offering a dual benefit of enhanced pest tolerance and potential growth support under pest pressure.

The combined treatment of MSNs and *T. absoluta* infestation (N/I) resulted in notable upregulation of key *JAZ* genes, suggesting that nanoparticle application primes the plant for an enhanced transcriptional response to pest stress. Such priming effects, where MSNs strengthen defense signaling and mitigate pest-induced suppression, are increasingly recognized in plant defense studies [59,61]. The observed upregulation of *SlJAZ19*, *SlJAZ20*, and *SlJAZ22* indicates that MSNs may enhance JA signaling responsiveness, effectively “priming” the plant for faster and stronger defense activation upon pest encounter. This finding aligns with research by Raliya, Nair, Chavalmane, Wang and Biswas [57], who reported that nanoparticles can trigger priming mechanisms, resulting in a more resilient plant response to subsequent stress.

The differential expression patterns of *SlJAZ* genes under the combined MSNs and *T. absoluta* treatment, as shown in Figure 10, suggest that members of the same gene family can play functionally distinct roles depending on environmental and stress cues. Similar divergence has been reported in other plant species—for instance, in *Arabidopsis*, AtJAZ1 and AtJAZ10 show opposite transcriptional responses under insect herbivory and hormonal treatments, indicating sub-functionalization within the gene family [47,62]. Likewise, in *Oryza sativa*, *OsJAZ* genes exhibit tissue-specific and stimulus-dependent expression divergence [63]. Such variation may arise from differences in promoter cis-regulatory elements, miRNA targeting, or protein–protein interactions within the JA signalling network. In tomatoes, the distinct cis-element profiles and predicted miRNA regulation of *SlJAZ* genes (Figure 6 and Supplementary Table S3) likely contribute to this heterogeneity. Moreover, nanoparticle exposure may alter hormonal sensitivity or redox balance, further influencing gene-specific expression.

Recent studies have highlighted the complexity of JAZ interactions with transcription factors in tomato defence against root-knot nematodes. For instance, Huang et al. [64] demonstrated that *SlWRKY45* physically interacts with multiple *SlJAZ* proteins, notably *SlJAZ1*, *SlJAZ2*, *SlJAZ7*, and *SlJAZ11*, and negatively regulates *Meloidogyne incognita* resistance by repressing JA biosynthesis through inhibition of *SlAOC* transcription. More recently, Huang et al. [65] revealed that *SlVQ15* recruits *SlWRKY30IIc*, which in turn binds the promoters of *SlJAZs* and represses their expression, establishing a regulatory loop in which *SlJAZ5* interferes with this interaction. These findings reflect intricate layers of feedback within JA signalling. Our current study complements these works by demonstrating that specific *SlJAZ* genes—such as *SlJAZ1*, *SlJAZ19*, and *SlJAZ22*—respond dynamically to MSNs and *T. absoluta* stress, suggesting that *JAZ-TF* complexes may also function under abiotic-biotic interaction contexts and could be sensitive to nanoparticle-based elicitors. Priming is a well-established concept in plant defense, typically induced by biological or chemical elicitors that “train” the plant to respond more effectively to future stresses [66]. In this study, MSNs appear to serve as abiotic priming agents, preparing the JA pathway to be more responsive to *T. absoluta* infestation. Such an effect has been documented with silicon nanoparticles, where pre-treated plants exhibit heightened defense responses when challenged by pests or pathogens [25,58,67]. Furthermore, the mitigating effect of MSNs under pest infestation suggests that these nanoparticles can enhance and sustain the plant’s innate defenses, thereby reducing the need for high pesticide inputs and offering a sustainable alternative that addresses environmental and health concerns [59]. Compared to previous studies that primarily focused on the structural identification and hormonal response of JAZ family members in tomato [49] or broadly characterized the JAZ role in jasmonate signalling [50], our study provides several novel insights. First, we present an updated and expanded genome-wide identification of 39 *SlJAZ* genes using the latest SL4.0 reference genome and domain-specific HMM profiling. Second, we integrate bioinformatics analyses with transcriptomic profiling under both mesoporous silica nanoparticles (MSNs) and *T. absoluta* stress conditions, offering the first exploration of nanoparticle-induced modulation of JAZ expression in tomato. Third, we report miRNA targeting predictions, protein–protein interaction networks, and syntenic relationships with other species, providing a multi-dimensional view of JAZ gene regulation.

In this study, synteny comparisons were made with *S. tuberosum* as the closest sequenced Solanaceae relative and *A. thaliana* as a widely used reference model. While these comparisons confirmed the expected evolutionary relationships, the inclusion of additional Solanaceae species such as *Capsicum annuum* or *Nicotiana tabacum* in future analyses would provide a more nuanced view of JAZ gene evolution across the family. Another limitation of this study is the absence of detailed phenotypic validation of tomato plants treated with MSNs under *T. absoluta* infestation. While our transcriptomic findings highlight potential roles of specific *SlJAZ* genes in JA-mediated defence, the lack of supporting physiological or agronomic data—such as pest damage assessment, plant growth measurements, or yield performance—prevents a full understanding of the functional impact. Future research should include comprehensive phenotypic analyses to confirm whether the transcriptional responses observed here translate into measurable resistance traits and improved plant performance in field conditions. The ability of MSNs to mitigate the adverse effects of *T. absoluta* infestation highlights their potential as a sustainable component of integrated pest management. By activating and priming natural defense pathways like the JA signaling cascade, MSNs offer a biologically favorable approach to pest control, potentially reducing pesticide reliance. This study’s findings contribute to a growing body of evidence suggesting that nanoparticle priming could be strategically implemented to strengthen crop resilience, thus paving the way for sustainable agricultural practices.

## 5. Conclusions

This study provides a comprehensive genome-wide characterization of the *SlJAZ* gene family in tomato and demonstrates their involvement in jasmonate-mediated defense responses under *T. absoluta* infestation and mesoporous silica nanoparticle (MSN) treatment. A total of 39 *SlJAZ* genes were identified, exhibiting diverse structural features, conserved motifs, and evolutionary conservation with orthologs in Arabidopsis and potato. Transcriptome profiling and qRT-PCR validation revealed that several *SlJAZ* genes, particularly *SlJAZ1*, *SlJAZ19*, *SlJAZ20*, and *SlJAZ22*, were induced under combined MSN and pest stress. Phenotypic assessments confirmed that MSN application reduced leaf damage, larval survival, and mining activity. Notably, regression analysis indicated that the expression of *SlJAZ20*, *SlJAZ26*, and *SlJAZ29* negatively correlated with pest-induced damage, suggesting their regulatory role in defense activation. Together, these findings establish MSNs as eco-compatible elicitors of JA-dependent immunity and identify specific *SlJAZ* genes as candidate markers for breeding insect-resistant tomato cultivars. The integration of nanotechnology, gene expression profiling, and phenotypic validation offers a promising strategy for enhancing plant resilience and reducing reliance on chemical pesticides in sustainable agriculture.

## Figures and Tables

**Table 1:** The duplication events for SlJAZ17-SlJAZ26.

Gene-1	Gene-2	Ka	Ks	Ka_Ks	Selection Pressure	Duplication Type	T = Ks/2r (MYA)
*SlJAZ12*	*SlJAZ21*	0.480957	1.872467	0.256858	Purifying	Segmental	144.0359
*SlJAZ38*	*SlJAZ20*	0.521719	N/A	N/A	N/A	Segmental	N/A
*SlJAZ38*	*SlJAZ29*	0.249556	1.284069	0.194348	Purifying	Segmental	98.77454
*SlJAZ19*	*SlJAZ20*	0.9835	N/A	N/A	N/A	Tandem	N/A
*SlJAZ18*	*SlJAZ25*	0.302624	0.860289	0.35177	Purifying	Segmental	66.17609
*SlJAZ17*	*SlJAZ26*	0.204479	0.567128	0.360552	Purifying	Segmental	43.62523
*SlJAZ27*	*SlJAZ35*	0.23627	0.725057	0.325864	Purifying	Segmental	55.77358

**Table 2:** Effects of Mesoporous Silica Nanoparticles (MSNs) and *T. absoluta* Infestation on Leaf Damage, Larval Survival, and Leaf Mining in Tomato Plants.

Treatment Group	Leaf Damage Index (%) (Mean ± SE)	Larval Survival Rate (%) (Mean ± SE)	Number of Leaf Mines per Leaf (Mean ± SE)
WN/WI (Control: No Pest, No MSNs)	0.0 ± 0.0 ^c^	-	0.0 ± 0.0 ^c^
WN/I (Pest Only)	45.2 ± 4.7 ^a^	84.6 ± 2.9 ^a^	12.4 ± 1.8 ^a^
N/WI (MSNs Only)	0.0 ± 0.0 ^c^	-	0.0 ± 0.0 ^c^
N/I (MSNs + Pest)	20.6 ± 3.8 ^b^	52.3 ± 4.4 ^b^	5.1 ± 1.2 ^b^

Note: Values represent mean ± standard error (SE). Different lowercase letters (a–c) within the same column indicate significant differences among treatments according to one-way ANOVA followed by Tukey’s HSD test (*p* < 0.05).

## Data Availability

The original contributions presented in this study are included in the article/Supplementary Materials. Further inquiries can be directed to the corresponding author.

## Supplementary Materials

The following supporting information can be downloaded at: https://www.mdpi.com/article/10.3390/insects16101046/s1, Supplementary Figure S1: Schematic of mesoporous silica nanoparticles (MSNs), Supplementary Table S1: Primers Used to study the expression of *SlJAZ* genes, Supplementary Table S2: Identified *JAZ* genes in the *S. lycopersicum* genome and their physicochemical properties, Supplementary Table S3: Top 12 identified conserved motif in SlJAZ proteins, Supplementary Table S4: Predicted miRNAs targeting *SlJAZ* genes, Supplementary Table S5: Regression analysis between the relative expression levels of qRT-PCR–validated *SlJAZ* genes and pest-related phenotypic parameters in tomato under different treatment conditions.

## Abbreviations

The following abbreviations are used in this manuscript:

JA	Jasmonic Acid
JAZ	Jasmonate ZIM-domain
MSNs	Mesoporous Silica Nanoparticles
qRT-PCR	Quantitative Real-Time Polymerase Chain Reaction
DEGs	Differentially Expressed Genes
SlJAZ	*Solanum lycopersicum* JAZ
GO	Gene Ontology
KEGG	Kyoto Encyclopedia of Genes and Genomes
miRNA	MicroRNA
LDI	Leaf Damage Index
LSR	Larval Survival Rate
LMN	Leaf Mine Number
R^2^	Coefficient of Determination
FDR	False Discovery Rate
ANOVA	Analysis of Variance
