# Supplementary material for: Genome-Wide Analysis and Functional Correlation of Tomato JAZ Genes Under Tuta absoluta Infestation and Nanoparticle-Induced Defense
Source: Insects. 2025 Oct 13;16(10):1046. doi: 10.3390/insects16101046 (PMC12564930; doi:10.3390/insects16101046)
Supplement: Supplementary file 1 [file insects-16-01046-s001.zip › Supplementary figure.docx]

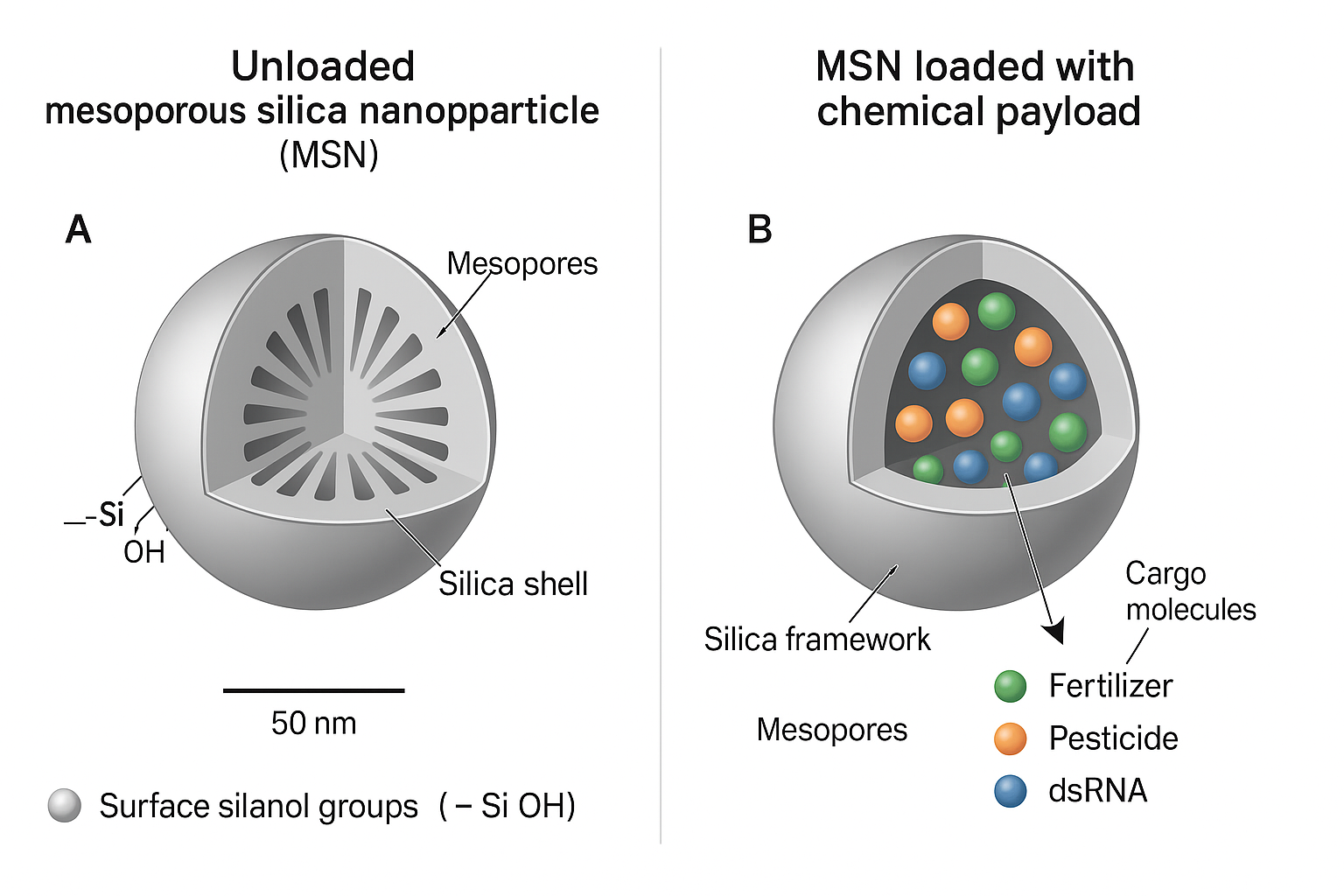


**Figure S1. Schematic of mesoporous silica nanoparticles (MSNs). Panel A – Unloaded MSN.** 3-D cut-away shows radial mesopores within the silica shell; surface silanol (-SiOH) groups are indicated. A 50 nm scale bar provides size context. **Panel B – MSN loaded with chemical payload.** Identical particle carrying representative cargo molecules—fertiliser (green), pesticide (orange), and dsRNA (blue)—inside the mesopores. Legend denotes colour code; labels highlight the silica framework and internal pore network.
